# Podemos Acreditar no Ecocardiograma de Rotina para Avaliar o Ventrículo Direito e a Insuficiência Pulmonar? Um Estudo Comparativo com Ressonância Magnética Cardíaca

**DOI:** 10.36660/abc.20200377

**Published:** 2021-10-06

**Authors:** Manuela Baima Cabral, Marcelo Felipe Kozak, Jorge Yussef Afiune

**Affiliations:** 1 Instituto de Cardiologia do Distrito Federal BrasíliaDF Brasil Instituto de Cardiologia do Distrito Federal – Cardiopediatria, Brasília, DF – Brasil

**Keywords:** Diagnóstico por Imagem, Imagem por Ressonância Magnética/métodos, Ecocardiografia/métodos, Função Ventricular Direita, Pneumopatias, Insuficiência Pulmonar, Cardiopatias Congênitas

## Abstract

**Fundamento:**

A ressonância magnética cardíaca (RMC) é o método de escolha para avaliar as dimensões e a função do ventrículo direito (VD), e a insuficiência pulmonar (IP).

**Objetivos:**

Avaliar a acurácia da ecocardiografia bidimensional (ECO 2D) em estimar a função e as dimensões do VD e o grau de IP, e comparar os resultados obtidos pela ECO 2D com os da RMC.

**Métodos:**

Comparamos os relatórios de ECO e RMC de pacientes cuja indicação para RMC havia sido para avaliar VD e IP. Um valor de p < 0,05 foi considerado estatisticamente significativo.

**Resultados:**

Incluímos 51 pacientes com cardiopatia congênita com idade mediana de 9,3 anos (7-13,3 anos). Observou-se uma baixa concordância entre ECO 2D e RMC quanto à classificação da dimensão (Kappa 0,19; IC 95% 0,05 a 0,33, p 0,004) e da função do VD (Kappa 0,16; IC 95% -0,01 a +0,34; p 0,034). O tamanho do VD foi subestimado pela ECO 2D em 43% dos casos, e a função do VD foi superestimada pela ECO 2D em 29% dos casos. O grau de concordância entre os métodos quanto à classificação da IP não foi significativo (Kappa 0,014; IC 95% -0,03 a +0,06; p 0,27). Houve uma tendência de a ECO 2D superestimar o grau da IP.

**Conclusões:**

A ECO 2D mostrou baixa concordância com a RMC quanto às dimensões e função do VD, e grau de IP. Em geral, a ECO subestimou as dimensões do VD e superestimou a função do VD e o grau de IP quando comparada à RMC.

## Introdução

O exame de ressonância magnética cardíaca (RMC) é considerado o método de escolha para avaliar as dimensões e a função do ventrículo direito (VD), bem como para quantificar insuficiência pulmonar (IP). Pacientes com cardiopatia congênita (CC) com envolvimento da via de saída do ventrículo direito (VD) ou da valva pulmonar estão sujeitos a desenvolverem dilatação e disfunção do VD e IP em algum estágio de suas vidas, resultante da história natural ou de procedimentos cirúrgicos ou hemodinâmicos. Nesses pacientes, a indicação da RMC é parte de uma abordagem “multi-imagem” (multi-imaging) recomendada atualmente.^1 , 2^ No entanto, a RMC é menos disponível que o ecocardiograma bidimensional (ECO 2D), principalmente em crianças, em quem sedação é comum. Assim, é fundamental otimizar as indicações da RMC e os encaminhamentos ao exame.

O ECO 2D, por sua vez, é um método amplamente disponível e, relativamente, de baixo custo, mas limitado quanto à avaliação do VD e do grau de IP.^3 - 10^ Apesar de tais limitações, na prática diária, a indicação da RMC é feita com base na avaliação de características clínicas e ecocardiográficas.

No presente estudo, nosso objetivo foi avaliar, retrospectivamente, a acurácia do ecocardiograma transtorácico bidimensional em estimar a função e as dimensões do VD e o grau de IP, e comparar seus resultados com os da RMC.

## Métodos

Este estudo foi conduzido em um centro de referência em tratamento de CC. A partir do um banco de dados de RMC, pediátrica/cardiopatias congênitas, selecionamos pacientes cuja indicação para o exame tinha sido avaliar o VD e a IP. Foram incluídos pacientes submetidos à RMC entre abril de 2015 e agosto de 2018. Comparamos laudos de ECO 2D e RMC mais próximos, quanto à data do exame, excluindo os pacientes que tivessem sido submetidos a algum tipo de tratamento invasivo entre os dois exames.^11^ O estudo foi aprovado pelo comitê de ética da instituição.

### Ecocardiograma bidimensional

Os exames foram realizados por cinco ecocardiografistas diferentes, treinados em cardiologia pediátrica e CC, com no mínimo de cinco anos de experiência, utilizando aparelho de ultrassom cardiovascular HD11 ou iE33 (Philips Medical Systems, Bothell, EUA). Todas as informações foram coletadas de laudos de ecocardiogramas, sem revisão das imagens ou análise dos métodos utilizados para conclusão do laudo. Em nosso serviço, o laudo é emitido apenas pelo médico que realiza o exame, sem a necessidade de sua revisão por outros médicos.

A análise subjetiva ou o escore-Z das medidas do VD obtidas no modo bidimensional (janela quatro câmaras) ou modo M (janela paraesternal) de acordo com as diretrizes da Sociedade Americana de Ecocardiografia são geralmente usadas para avaliar as dimensões do VD.^12 , 13^ Em nosso serviço, os principais métodos para avaliar a função do VD são: análise qualitativa (visual), TAPSE (excursão sistólica do plano do anel tricúspide do laudo), fração de variação da área do VD (FAC, *fractional area change* ), e pico de velocidade da onda S´ por Doppler tecidual pulsado (DTP). Tanto o TAPSE como o DTP são também apresentados em escore-Z.^14 , 15^ O conjunto desses parâmetros determina a impressão final do examinador. Para a avaliação da IP, os seguintes parâmetros são geralmente utilizados: diâmetro do jato regurgitante/diâmetro da via de saída do VD, local no tronco da artéria pulmonar onde o refluxo é detectado, meio-tempo de pressão da curva de fluxo e integral da velocidade-tempo (IVT). Após essa análise, o examinador utiliza a estratificação em quatro níveis para classificação da função do VD (normal, ou disfunção leve, moderada ou grave), tamanho do VD (normal, ou dilatação leve, moderada ou grave), e grau de IP (ausente, ou insuficiência leve, moderada ou grave). A função do ventrículo esquerdo foi analisada pela fração de ejeção (FE) obtida pelo método de Teichholz e/ou Simpson.

### Ressonância magnética cardíaca

Os exames de RMC foram realizados em um aparelho 1.5 Tesla (SIGNA®, General Electric). Os volumes ventriculares foram determinados após a aquisição de imagens bidimensionais pela técnica de precessão livre em estado de equilíbrio (SSFP *steady state free precession* ) no eixo curto do coração ou corte axial do tórax, desde o anel atrioventricular até o ápice do coração. Os volumes ventriculares e a FE foram determinados após o traçado manual em modo *offline* utilizando o sistema de análise MASS R MR Analytical Software System, versão 5.1 (Leiden University Medical Center and MEDIS Medical Imaging system). As imagens foram obtidas a partir dos pacientes em respiração livre, com número de excitações (NEX) de três, ou em apneia (NEX de 1), dependendo da situação clínica do paciente e qualidade das imagens. Foram avaliados o volume diastólico final do VD indexado (VDFVDi), a FE do VD, o volume sistólico final do VD indexado (VSFVDi) e a FE do VE.

Os volumes ventriculares indexados foram transformados em escore-Z, com base nos valores normais publicados pela Sociedade de Ressonância Magnética Cardiovascular ( Tabela 1 ),^16^ utilizando-se a seguinte fórmula: escore-Z = [medida do paciente – média esperada)/desvio padrão (DP)]. Para os pacientes com idade inferior a oito anos, os cálculos foram realizados com base no estudo de Valsangiacomo-Buechel ( Tabela 2 ),^17^ utilizando a fórmula: Z = log10 (medida/esperada)/DP (DP = 0,05). Um escore-Z entre -2 e +2 foi considerado normal; um escore-Z entre +2,01 e +3 uma dilatação leve; um escore-Z entre +3,01 e +4 dilatação moderada, e um escore-Z > +4 considerado como dilatação grave.

**Tabela 1:** – Valores normais para volumes ventriculares indexados em adultos e crianças maiores de oito anos de idade

	Adultos (> 18 anos)		Crianças (8-17 anos)	

	Homens	Mulheres	Homens	Mulheres
**VDFVDi (mL/m** ^**2**^ **)**	81 ± 12	76 ± 10	80 ± 11	75 ± 11
**VSFVDi (mL/m** ^**2**^ **)**	91 ± 15	80 ± 16	84 ± 10	76 ± 10

*VDFVDi: volume diastólico final do ventrículo direito indexado; VSFVDi: volume sistólico final do ventrículo direito indexado.*

**Tabela 2:** – Valores normais de volume ventricular para crianças menores de oito anos de idade

	Crianças (< 8 anos)	

	Meninos	Meninas
**VDFVE (mL)**	77,5xSC^1.38^	67,8xSC^1.38^
**VDFVD (mL)**	83,8xSC^1.47^	72,7xSC^1.47^

*VDFVE: volume diastólico final do ventrículo esquerdo; VDFVD: volume diastólico final do ventrículo direito.*

A classificação da FE foi realizada da seguinte maneira:

- Ventrículo direito: FE> 45% = normal; FE de 36 a 45% = disfunção leve; FE de 25 a 35% = disfunção moderada; FE < 25% = disfunção acentuada.
- Ventrículo esquerdo: FE > 50% = normal; FE de 40 a 50% = disfunção leve; FE de 30 a 39% = disfunção moderada; FE < 30% = disfunção acentuada.

A IP foi avaliada por imagens de contraste de fases, adquiridas perpendicularmente ao tronco e aos ramos pulmonares, em respiração livre (NEX de 2 ou 3), ajustando-se o número de visualizações por segmento de acordo com a frequência cardíaca do paciente, permitindo a reconstrução de 30 fases por intervalo RR. O volume e a fração de regurgitação pulmonar foram analisados pelo programa FLOW R MR Flow Quantification software, versão 3.1 (MEDIS Medical Imaging system and Leiden University Medical Center). Como existem dois métodos possíveis para classificar o grau de IP por RMC (volume regurgitante e fração de regurgitação), divergências entre as classificações foram resolvidas pela análise do examinador. Os seguintes valores foram considerados para classificação:

- IP leve: volume regurgitante = 0,3-1,0 L/min/m^2^ ou fração de regurgitação = 5-20%.
- IP moderada: volume regurgitante =1,01-2,50 L/min/m^2^ ou fração de regurgitação = 21-40%.
- IP acentuada: volume regurgitante > 2,5 L/min/m^2^ ou fração de regurgitação > 40%.

### Análise estatística

As variáveis contínuas foram expressas em média ± desvio padrão ou mediana e intervalo interquartil (25-75), dependendo da distribuição dos dados. A distribuição dos dados pode ser considerada normal quando aproximadamente 95% dos dados encontram-se dentro de 1,96 desvios padrões da média (bilateralmente). As variáveis categóricas foram apresentadas em números absolutos e porcentagens. A análise de concordância foi realizada usando o coeficiente Kappa, com auxílio do programa StatsDirect, versão 2.7.2, 2008 (Cheshire, UK). A significância estatística foi considerada como p < 0,05 (teste bilateral).

## Resultados

Do total de 178 crianças e adultos com CC submetidos à RMC durante o período do estudo, 51 (29%) foram incluídos na análise. As características principais da população estudada estão descritas na Tabela 3 .

**Tabela 3:** – Características da população estudada (n = 51) e seus diagnósticos

Características	
Sexo masculino	30 (58%)
Peso (Kg) (DP)	33.0 ± 16.7
Área de superfície corporal (m^2^) (IIQ)	1.0 (0.8-1.4)
Idade na ocasião do tratamento cirúrgico (anos) (IIQ)	1.3 (0.8-2.4)
Idade no dia da RMC (anos) (IIQ)	9.2 (6.9-13.3)
Idade no dia do ecocardiograma (anos) (IIQ)	9.0 (6.8-13.0)
Tempo entre tratamento cirúrgico e RMC (anos) (IIQ)	7.2 (5.4-11.1)
Intervalo entre RMC e ecocardiograma (dias) (IIQ)	124 (70-188)
**Diagnósticos principais**	
Tetralogia de Fallot	35 (68%)
Estenose da valva pulmonar	6 (12%)
Tronco arterial comum	3 (6%)
Atresia pulmonar com comunicação interventricular	2 (4%)
Tetralogia de Fallot com agenesia da valva pulmonar	2 (4%)
Insuficiência pulmonar congênita	1 (2%)
Defeito do septo atrioventricular com estenose pulmonar	1 (2%)
Atresia pulmonar com septo interventricular intacto	1 (2%)

*IIQ: intervalo interquartil; RMC: ressonância magnética cardíaca.*

### Achados ecocardiográficos

As dimensões do VD foram classificadas como anormais em 96% dos casos, com dilatação leve em 16% dos casos, dilatação moderada em 43%, e dilatação acentuada em 37%. Caso os ecocardiografistas tivessem considerado apenas o escore-Z do diâmetro diastólico final do VD o (DDFVD), haveria uma piora na classificação em 42 pacientes (82%), nenhuma alteração em cinco pacientes (10%), e uma classificação melhor ou mais apropriada em quatro pacientes (8%) em comparação à RMC. A disfunção ventricular direita foi encontrada em 20% dos casos, sendo leve em 10%, moderada em 8% e acentuada em 2%. Dos 51 pacientes, 29 (57%) possuíam dados disponíveis de TAPSE, FAC, e DTP em seus lados e, desses 29 pacientes, 18 (62%) apresentavam valores abaixo do intervalo normal. Apesar desse resultado, nove desses 18 pacientes foram classificados como tendo função sistólica do VD normal. A concordância entre esses índices ecocardiográficos da função sistólica do VD e da FE do VD na RMC foi de 38%. Se o ecocardiografista tivesse considerado apenas esses índices anormais, sem considerar o exame visual (método *eyeball* ), haveria erros de classificação em mais nove casos e uma classificação correta em mais cinco casos. As medidas ecocardiográficas são apresentadas na Tabela 4 . A IP foi leve em 2%, moderada em 2% e grave em 94%; em um caso, a IP não foi classificada. Em nenhum dos casos foi observada dilatação sistólica ou disfunção do VE ( Tabela 5 ).

**Tabela 4:** – Medidas ecocardiográficas dos pacientes estudados

Parâmetros	Valores
Diâmetro diastólico final do VD (mm)	30,5 ± 7,48
Escore-Z do diâmetro diastólico final do VD	2 (1,48-2,53)
TAPSE (mm) (n = 15)	17,9 ± 3,59
Escore-Z da TAPSE	-2,3 (-3,28 a -0,94)
FAC (%) (n = 7)	41 ± 12,3
Pico S´ VD cm/s (n = 23)	9,1 ± 1,89
Escore-Z do pico S´ VD cm/s	-2,4 (-3,22 a -1,69)
Diâmetro do VD / diâmetro do VE (mm)	0,8 (0,71-0,99)

*VD: ventrículo direito; TAPSE: excursão sistólica do plano do anel tricúspide (tricuspid annular plane systolic excursion), FAC: fração de variação da área do VD (fractional area change); S´ VD: pico de velocidade da onda S.*

**Tabela 5:** – Comparação entre achados do ecocardiograma bidimensional (2D ECO) e da ressonância magnética cardíaca (RMC)

			Normal	Dilatação leve	Dilatação moderada	Dilatação grave
DIMENSÃO	VD	ECO	2 (4%)	8 (16%)	22 (44%)	19 (38%)
		RMC	3 (6%)	3 (6%)	6 (12%)	39 (76%)
	VE	ECO	51 (100%)	0	0	0
		RMC	38 (76%)	5 (10%)	3 (6%)	5 (10%)
			**Normal**	**Disfunção leve**	**Disfunção moderada**	**Disfunção grave**
FUNÇÃO	VD	ECO	41 (80%)	5 (10%)	4 (8%)	1 (2%)
		RMC	30 (59%)	15 (29%)	4 (8%)	2 (4%)
	VE	ECO	51 (100%)	0	0	0
		RMC	35 (69%)	14 (27%)	1 (2%)	1 (2%)
			**Ausente**	**Leve**	**Moderada**	**Grave**
INSFUCIÊNCIA PULMONAR		ECO	0	1 (2%)	1 (2%)	48 (96%)
		CMR	1 (2%)	8 (16%)	32 (64%)	9 (18%)

*VD: ventrículo direito; VE: ventrículo esquerdo.*

### Achados da RMC

O exame foi realizado sob anestesia em 16 pacientes (31%). O VDFVDi variou entre 84,7 e 275,6mL/m^2^, o que correspondeu a um escore-Z médio de +5.6 ± 2. Três pacientes (6%) apresentaram escore-Z do VDFVDi dentro dos limites normais (91,4 ± 7,9 mL/m^2^), três (6%) apresentaram dilatação leve (110,6 ± 4,8 mL/m^2^), seis (12%) apresentaram dilatação moderada (120 ± 10,9 mL/m^2^) e 39 (76%) dilatação grave (151,8 ± 31,5 mL/m^2^). O VDFVEi variou entre 66,4 e 155,2 mL/m^2^ ( Tabela 6 ). Observou-se dilatação do ventrículo esquerdo em 25% dos pacientes e disfunção sistólica do ventrículo esquerdo em 31% ( Tabela 5 ). Dos 16 pacientes com disfunção do ventrículo esquerdo, o exame foi realizado sob anestesia em 12 (75%).

**Tabela 6:** – Principais achados da ressonância magnética cardíaca nos pacientes estudados (n=51)

Parâmetros analisados	Valores
Volume diastólico final do VD indexado (mL/m^2^)	142,1 ± 33,4
volume sistólico final do VD indexado (mL/m^2^)	76,1 ± 27,4
Fração de ejeção do VD (%)	46,9 ± 10,2
Insuficiência pulmonar (L/min/m^2^)	1,5 (1,01-1,99)
Insuficiência pulmonar (%)	31,1 ± 10,7
Volume diastólico final do VE indexado (mL/m^2^)	87,4 ± 18,5
Volume sistólico final do VE indexado (mL/m^2^)	42,4 ± 12,8
Fração de ejeção do VE (%)	53,6 ± 8,4%
Volume do VD / Volume do VE	1,6 (1,43-1,86)

*Volume do VD / Volume do VE*

## Análise de concordância entre ECO 2D e RMC

### Dimensão do ventrículo direito

**Figura 1:**
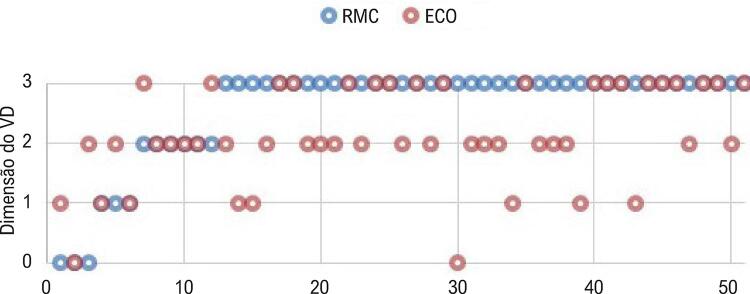
– *Gráfico comparando a classificação da dimensão do VD por RMC e ECO para cada paciente.*

Observou-se uma concordância significativa entre os métodos quanto à classificação das dimensões do ventrículo direito (Kappa 0,19; IC95% 0,05 a 0,33, p 0,004), apesar do baixo grau de concordância. A diferença na classificação foi de um grau em 74% dos casos discordantes, com subestimação das dimensões do VD pelo ECO em 22 casos (43%), e superestimação em cinco casos (10%) ( Figura 1 ). Dos 39 casos de dilatação grave, a ecocardiografia realizou um diagnóstico correto em 17 (44%). A sensibilidade do ECO em detectar casos moderados e graves de dilatações moderadas ou acentuadas do VD foi de 87%, e a especificidade de 67%.

### Função sistólica do VD

**Figura 2:**
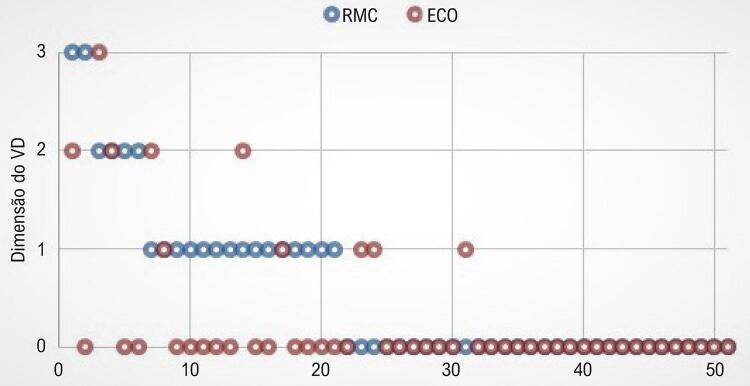
– *Gráfico comparando a classificação da função sistólica do VD por RMC e ECO para cada paciente.*

Observou-se uma fraca concordância entre os métodos para a classificação da função do VD (Kappa 0,16; IC95% -0,01 a +0,34; p 0,034). A diferença na classificação foi de um grau em 86% dos casos discordantes, com superestimação da função do VD pela ecocardiografia em 15 casos (29%), e subestimação em seis casos (12%) ( Figura 2 ). Os dois casos de disfunção grave do VD não foram detectados pelo ECO, sendo um foi classificado como normal e o outro como disfunção moderada.

### Insuficiência pulmonar

**Figura 3:**
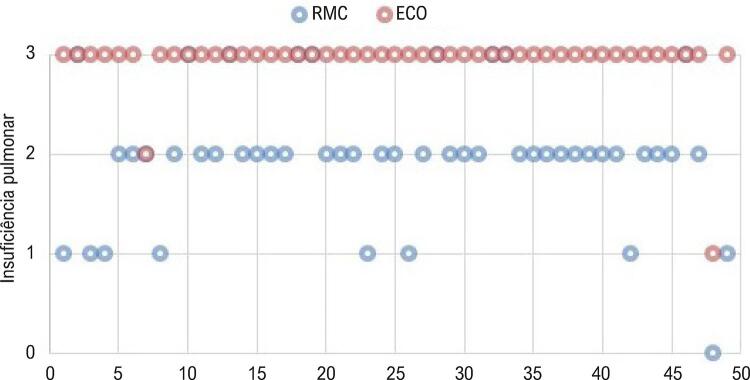
– *Gráfico comparando a classificação da insuficiência pulmonar pela RMC e ECO para cada paciente.*

O grau de concordância entre os métodos quanto à classificação de IP não foi estatisticamente significativo (Kappa 0,014; IC95% -0,03 a +0,06, p 0,27). Houve discordância em 80% dos casos, com uma tendência de superestimação do grau de IP pelo ECO. Houve discordância superior a um grau em 20% dos casos ( Figura 3 ).

### Função sistólica e dimensão do VE

O ECO não identificou nenhum dos 16 casos (31%) de disfunção do VE e nenhum dos 13 casos (25%) de dilatação do VE, o que não permitiu a avaliação da concordância entre os métodos. Dos 16 casos de disfunção do VE, 10 (62%) também apresentaram disfunção do VD e somente um apresentou VD com dimensões normais.

## Discussão

A IP residual e remodelação do VD ao longo do tempo são elementos importantes na evolução tardia de pacientes que se submeteram à correção cirúrgica da via de saída do VD na infância. A tomada de decisão quanto ao tempo de se realizar uma nova intervenção cirúrgica nesses casos é desafiadora. Os critérios clínicos, eletrocardiográficos, além de achados de exames de imagens, tais como ECO e RMC, são utilizados para ajudar nessa decisão, buscando determinar o melhor momento para o tratamento cirúrgico.

O ECO 2D é o método de imagem mais utilizado para monitorar a evolução desses pacientes e orientar, na maioria das vezes, a tomada de decisão. Dadas as dificuldades em se realizar a avaliação do VD por ECO 2D com parâmetros objetivos, a avaliação qualitativa (visual, ou *eyeball* ) das dimensões e da função do VD é ainda muito utilizada. No entanto, a acurácia dessa avaliação é questionável e ainda fomenta mais estudos. Nosso estudo objetivou avaliar a acurácia do ECO 2D, realizado na rotina ambulatorial, em quantificar as dimensões e a função do VD e o grau de IP. Escolhemos pacientes com CC com risco de desenvolverem dilatação e disfunção do VD, bem como IP.

Observamos certa concordância, embora fraca, entre ECO e RMC, para a classificação das dimensões do VD. Esse resultado é similar ao encontrado por Lai et al.,^4^ quando aplicaram as recomendações para avaliação do VD utilizando ECO 2D em pacientes com parâmetros normais, em pacientes com defeitos do septo atrial e em pacientes que se submeteram ao tratamento cirúrgico da tetralogia de Fallot. Em seu artigo, os autores sugerem que a imprecisão relacionada ao ECO 2D é inerente à aplicação das medidas lineares ou das áreas em uma câmara de geometria complexa. Puchalski et al.,^18^ em um estudo com um delineamento similar ao nosso, obtiveram resultados similares e enfatizaram que a elevada variabilidade entre observadores é uma fonte potencial de discordância entre ECO e RMC. Sugere-se que a adição de medidas objetivas às medidas subjetivas aumentaria a concordância entre os métodos.^7 , 19^ Nesse sentido, Greutmann et al.^20^ observaram, em uma população adulta, que a medida linear com melhor correlação com o volume diastólico do VD foi a área do VD em um corte apical de quatro câmaras. A partir dessa medida, Alghandi et al.^21^ conseguiram discriminar pacientes com VDFVDi > 170 mL/m^2^. Estudos mais recentes têm investigado o uso da ecocardiografia tridimensional (3D ECO) para avaliar o VD e, mesmo assim, geralmente observa-se subestimação das medidas das dimensões do VD,^7 , 22 - 24^ é explicada por sua incompleta visualização, especialmente em casos de dilatação acentuada do VD, quando o ápice cardíaco está deslocado e tem difícil acesso no exame ecocardiográfico. Em nosso estudo, apesar de termos analisado uma população jovem, 86% dos pacientes apresentaram dilatação moderada ou grave do VD. Tal fato pode explicar a falta de acurácia, se considerarmos que quanto maior o VD, maiores são as dificuldades em avaliá-lo. Contudo, em nosso estudo, o ECO 2D foi capaz de detectar 96% dos casos de dilatação moderada e/ou grave, o que justificaria o rastreamento de pacientes que necessitaria ser submetido a um exame de RMC. Em relação à função sistólica do VD, nossos resultados estão de acordo com aqueles apresentados por Puchalski,^18^ que demonstrou um baixo grau de concordância entre ECO 2D e RMC. No entanto, o grau de discordância entre as classes de estratificação foi de apenas um grau em 86% dos casos, e essa diferença ocorreu principalmente na zona entre disfunção leve e função normal. Vários métodos ecocardiográficos têm sido usados para avaliar a função do VD, incluindo TAPSE, FAC, DTP, entre outros. Apesar de alguns serem reprodutíveis, nenhum mostrou uma forte correlação com a FE do VE na população pediátrica, especialmente em pacientes com DCC.^3 , 7 - 10 , 25 - 27^ Métodos que utilizam medidas unidimensionais, tais como TAPSE, ou medidas bidimensionais tais como a fração de variação da área do VD, não levam em consideração a complexa geometria do VD. Esses dois métodos não consideram, por exemplo, a via de saída do VD, comumente comprometida em pacientes submetidos à cirurgia de correção da tetralogia de Fallot^21^ Ainda, outros fatores influenciam essas medidas, tais como pericardiectomia, pré-carga, ângulo do feixe de ultrassom, qualidade da janela acústica, função do VE, entre outros. No estudo de Mercer-Rosa et al.^16^ TAPSE não se correlacionou com a FE do VD por RMC ou com o pico de VO_2_. Os mesmos autores, em 2012, observaram uma fraca correlação entre Doppler tecidual da parede livre do VD e a FE do VD por RMC, e a ausência de correlação entre o índice de Tei e a FE do VD por RMC em pacientes submetidos ao tratamento cirúrgico para tetralogia de Fallot.^5^ Em um estudo de Leong et al.^28^ com uma população adulta com insuficiência cardíaca, a avaliação da função do VD pelo ECO com *speckle tracking* apresentou a correlação mais forte (r = 0.77) com a FE do VD na RMC, em comparação a outras técnicas como TAPSE, FAC e DTP. No entanto, deve-se considerar que essa população geralmente não se apresente com dilatação grave do VD. A ECO 3D talvez seja o método que melhor se correlacione com a RMC,^29^ embora esteja longe de se tornar tão disponível como a ECO 2D, principalmente na população jovem.^30^ Na prática, muitos serviços optam pela avaliação qualitativa ( *eyeball* ) da função do VD, combinada com alguns outros índices mencionados anteriormente para corroborarem a impressão final. Assim, a avaliação da função do VD por ECO 2D torna-se um método dependente do examinador e sujeito a diferentes interpretações.

Em relação ao grau de IP, não encontramos concordância entre ECO e RMC. Em 38 pacientes (75% da amostra), a IP foi classificada como acentuada pelo ECO, mas como moderada ou leve pela RMC. Em outras palavras, ECO superestimou o grau de IP na maioria dos casos, resultado similar ao observado por Mercer-Rosa et al.^5^ Em seu estudo,^5^ muitos pacientes com IP leve pela RMC foram classificados como IP moderada ou acentuada pela ECO, utilizando a razão IVTdiastólica/IVT sistólica no tronco pulmonar. Renella et al.,^31^ observaram que o ECO apresentou elevada sensibilidade em identificar casos de IP grave em diferentes métodos utilizados, e recomendaram a avaliação de curvas Doppler nos ramos pulmonares, em vez do tronco pulmonar, a fim de aumentar a especificidade do método. Na prática, a literatura tem mostrado que o ECO é capaz de discriminar casos de IP leve dos casos moderados a grave, o que é corroborado por nossos resultados.

A disfunção do VE tem sido descrita em pacientes submetidos a reparo de doenças do VD e associada a eventos adversos maiores.^32 - 37^ Assim, a avaliação do VE não pode ser negligenciada. Em nossa amostra, a ECO 2D mostrou função normal do VE em todos os pacientes, enquanto a RMC identificou disfunção em 31% dos casos, o que mostra limitações do 2D ECO em avaliar a função ventricular esquerda nessa situação. Dos pacientes com disfunção ventricular esquerda. Os pacientes com disfunção do VE, 62% também apresentaram algum grau de disfunção do VD, o que pode estar relacionado com interdependência ventricular.^38 , 39^ A nosso ver, a avaliação da função do VE em casos de dilatação ou disfunção do VD é mais difícil para o ECO devido ao movimento paradoxal do septo interventricular e deslocamento do VE por um VD dilatado ou disfuncional, o que torna sua visibilidade mais difícil.

### Limitações do estudo

Esse foi um estudo retrospectivo, unicêntrico, com um número limitado de pacientes. A possibilidade de viés de inclusão deve ser considerada, uma vez que a decisão de submeter o paciente à RMC é geralmente feita após análise dos achados ecocardiográficos. A análise dos resultados do ECO foi feita com base nos laudos somente, sem revisão das imagens. Considerando que os exames de ECO foram realizados por cinco ecocardiografistas diferentes, existe a possibilidade de discordâncias entre observadores e intraobservador. Ainda, o intervalo de tempo entre o ECO e a RMC foi relativamente grande. Apesar de nenhum procedimento cirúrgico ou percutâneo tenha sido realizado entre o ECO e a RMC, o ideal seria que os exames tivessem sido realizados dentro de 24 horas entre eles. O uso do escore-Z das medidas ventriculares obtidas por RMC não é uma abordagem comum na literatura, e o intervalo de classificação, bem como o impacto clínico dessas medidas, precisam ser mais bem avaliados em estudos futuros

## Conclusões

Em nossa prática e, considerando as limitações do estudo, o 2D ECO mostrou uma baixa concordância com a RMC quanto às dimensões e função sistólica do VD, bem como ao grau da IP. Em geral, o ECO subestimou as dimensões do VD e superestimou a função do VD e o grau de IP. Acreditamos que a incorporação de parâmetros ecocardiográficos mais objetivos seja necessária para uma melhor concordância com a RMC. No entanto, o impacto real dessa análise em relação à tomada de decisão clínica ainda é incerto.
