# Supplementary material for: Podemos Acreditar no Ecocardiograma de Rotina para Avaliar o Ventrículo Direito e a Insuficiência Pulmonar? Um Estudo Comparativo com Ressonância Magnética Cardíaca
Source: Arq Bras Cardiol. 2021 Oct 6;117(4):690–8. [Article in Portuguese] doi: 10.36660/abc.20200377 (PMC8528354; doi:10.36660/abc.20200377)
Supplement: Translation

# Translation: Can we Trust in Routine Echocardiography to Assess the Right Ventricle and Pulmonary Insufficiency? A Comparative Study with Cardiac Magnetic Resonance

**Authors:** Manuela Baima Cabral, Marcelo Felipe Kozak, Jorge Yussef Afiune

**Affiliations:** 1 Brazil Instituto de Cardiologia do Distrito Federal – Cardiopediatria, Brasília, DF – Brazil

**Keywords:** Diagnostic, Imaging, Magnetic Resonance, Imaging, Echocardiography/methods, Ventricular Function, Right, Lung Diseases, Pulmonary Valve, Insufficiency, Heart Defects, Congenital

## Abstract

**Background:**

Cardiac magnetic resonance (CMR) is the method of choice for assessing right ventricular (RV) dimensions and function, and pulmonary insufficiency (PI).

**Objectives:**

To assess the accuracy of two-dimensional echocardiography (2D ECHO) in estimating RV function and dimensions, and the degree of PI, and compare the 2D ECHO and CMR findings.

**Methods:**

We compared ECHO and CMR reports of patients whose indication for CMR had been to assess RV and PI. A p-value < 0.05 was considered statistically significant.

**Results:**

We included 51 congenital heart disease patients, with a median age of 9.3 years (7-13.3 years). There was poor agreement between 2D ECHO and CMR for classification of the RV dimension (Kappa 0.19; 95% CI 0.05 to 0.33, p 0.004) and function (Kappa 0.16; 95% CI -0.01 to +0.34; p 0.034). The RV was undersized by 2D ECHO in 43% of the cases, and RV function was overestimated by ECHO in 29% of the cases. The degree of agreement between the methods in the classification of PI was not significant (Kappa 0.014; 95% CI -0.03 to +0.06, p 0.27). 2D ECHO tended to overestimate the degree of PI.

**Conclusions:**

The 2D ECHO showed a low agreement with CMR regarding the RV dimensions and function, and degree of PI. In general, ECHO underestimated the dimensions of the RV and overestimated the function of the RV and the degree of PI as compared with CMR.

## Introduction

Cardiac magnetic resonance imaging (CMR) is considered the method of choice to assess dimensions and function of the right ventricle (RV), as well as to quantify pulmonary insufficiency (PI). Patients with congenital heart disease (CHD) and involvement of the right ventricular outflow tract or pulmonary valve are subject to develop dilation and dysfunction of the RV and PI at some stage in their lives, as a result of either the natural history or surgical or hemodynamic procedures. In this type of patient, indication for CMR is part of the multi-imaging approach recommended today.^1 , 2^ However, CMR is less available than two-dimensional echocardiogram (2D ECHO), especially for young children, when sedation is frequent. Thus, it is crucial to optimize CMR indications and referrals for CMR.

In turn, 2D ECHO is a widely available and relatively low-cost method but limited in assessing the RV and the degree of PI.^3 - 10^ Despite this, in daily practice, indication for CMR is made based on the evaluation of clinical and echocardiographic features.

In our study, we sought to retrospectively assess the accuracy of transthoracic 2D ECHO in estimating the function and dimensions of the RV and the degree of PI, comparing its findings with those of CMR.

## Methods

This study was carried out in a reference center for the treatment of CHD. We selected, from the pediatric/congenital CMR database, patients whose indication for CMR had been to assess the RV and PI. Patients who underwent CMR between April 2015 and August 2018 were included. We compared the closest (in terms of the date of the tests) 2D ECHO and CMR reports, excluding patients who underwent some type of invasive treatment between the two tests.^11^ This study was approved by the institutional review board.

### Two-dimensional echocardiogram

The examinations were performed by five different echocardiographers, trained in pediatric cardiology and CHD, with at least five years of experience, using either HD 11 or iE33 cardiac ultrasound systems (Philips Medical Systems, Bothell, USA). All information was collected from ECHO reports, with no review of the images or analysis of the methods to conclude the report. In our service, the report is issued only by the performing physician, and there is no mandatory review of the reports by other physicians.

Subjective analysis or Z-score of measurements of the RV obtained in two-dimensional mode (four-chamber window) or M-mode (parasternal window) according to the American Society of Echocardiography guidelines are generally used to assess right ventricular dimensions.^12 , 13^ In our service, the main methods to assess the function of the RV are: qualitative analysis (eyeball method), TAPSE (tricuspid annular plane systolic excursion), fractional area change (FAC) and the S’ wave peak systolic velocity by pulsed-wave tissue Doppler imaging (TDI). TAPSE and RV TDI s´ are also plotted in Z-scores.^14 , 15^ The set of these parameters determines the examiner's final impression. To assess PI, the following parameters are generally used: diameter of the regurgitant jet / diameter of the RV outflow tract, location of the pulmonary artery tree where reflux is detected, pressure half-time of the flow curve and integral velocity-time (IVT). After this analysis, the examiner uses a four-level stratification to classify right ventricular function (normal, or mild, moderate, or severe dysfunction), size of the RV (normal, or mild dilation, moderate, or severe dilation) and the degree of PI (absent, or mild, moderate, or severe insufficiency). For analysis of the left ventricular (LV) function, the ejection fraction (LVEF), obtained by the Teichholz and/or Simpson method, was used.

### Cardiac magnetic resonance

The CMR examinations were carried using a 1.5 Tesla scanner (General Electric SIGNA^TM^). Ventricular volumes were determined after acquisition of two-dimensional images in cine-SSFP (steady state free precession) in the short axis of the heart or axial section of the chest, from the atrioventricular ring to the cardiac apex. Ventricular volumes and EF were determined after manual tracing in offline analysis using the MASS R MR Analytical Software System, version 5.1 (Leiden University Medical Center and MEDIS Medical Imaging system). The images were obtained in free breathing with number of excitations (NEX) of three, or in apnea (NEX of 1), depending on patient's clinical situation and quality of the images. The indexed end diastolic volume of the RV (RVEDVi), the right ventricular EF, the indexed end diastolic volume of the left ventricle (LVEDVi) and the LVEF were evaluated.

Indexed ventricular volumes were transformed into a Z-score, based on the normal values published by the Society for Cardiovascular Magnetic Resonance ( Table 1 ),^16^ using the following formula: Z-score = (patient measurement – expected average)/standard deviation [SD]). For patients younger than eight years old, calculations were made based on the study by Valsangiacomo-Buechel ( Table 2 ),^17^ using the formula: Z = log10 (measured/expected)/SD (SD = 0.05). A Z-score between -2 and +2 was considered normal; a Z-score between +2.01 and +3 a mild dilation; a Z-score between +3.01 and +4 moderate dilation, and a Z-score > +4 was considered severe dilation.

**Table 1:** – Values for normal indexed ventricular volumes in adults and children older than eight years of age

	Adults (> 18 years)		Children (8-17 years)	

	Male	Female	Male	Female
**LVEDVi (ml/m** ^**2**^ **)**	81 ± 12	76 ± 10	80 ± 11	75 ± 11
**RVEDVi (ml/m** ^**2**^ **)**	91 ± 15	80 ± 16	84 ± 10	76 ± 10

*LVEDV: left ventricular end-diastolic volume; RVEDV: right ventricular end diastolic volume.*

**Table 2:** – Values for normal ventricular volumes in children under 8 years of age

	Children (< 8 years)	

	Boys	Girls
**LVEDV (mL)**	77.5xBSA^1.38^	67.8xBSA^1.38^
**RVEDV (mL)**	83.8xBSA^1.47^	72.7xBSA^1.47^

*BSA: body surface area; LVEDV: left ventricular end-diastolic volume; RVEDV: right ventricular end diastolic volume.*

Regarding EF classification, it was performed as follows:

- Right ventricle: EF > 45% = normal; EF from 36 to 45% = mild dysfunction; EF from 25 to 35% = moderate dysfunction; EF < 25% = severe dysfunction.
- Left ventricle: EF > 50% = normal; EF from 40 to 50% = mild dysfunction; EF from 30 to 39% = moderate dysfunction; EF < 30% = severe dysfunction.

PI was evaluated by phase contrast imaging performed perpendicular to the pulmonary trunk and perpendicular to the pulmonary branches, in free breathing (NEX of 2 or 3), adjusting the number of views per segment according to patient's heart rate, allowing the reconstruction of 30 phases per RR interval. Pulmonary regurgitation volume and fraction were analyzed using the FLOW R MR Flow Quantification software, version 3.1 (MEDIS Medical Imaging system and Leiden University Medical Center). As there are two possible methods to classify the degree of PI by CMR (regurgitant volume and fraction of regurgitation), discrepancies were resolved by analysis of the data by the observer. The following values were considered for classification:

- Mild PI: regurgitant volume = 0.3-1.0 L/min/m^2^ or regurgitation fraction = 5-20%.
- Moderate PI: regurgitant volume = 1.01-2.50 L/min/m^2^ or regurgitation fraction = 21-40%.
- Severe PI: regurgitant volume > 2.5 L/min/m^2^ or regurgitation fraction > 40%.

### Statistical analysis

Continuous variables were expressed as mean ± standard deviation or median with interquartile range (25-75), depending on data distribution. Data distribution can be considered normal when around 95% of the data fall within 1.96 standard deviations from the mean (bilaterally). Categorical variables were presented in absolute numbers and percentages. Agreement was analyzed using the Kappa coefficient, with the aid of the StatsDirect program, version 2.7.2, 2008 (Cheshire, UK). Statistical significance was considered as p < 0.05 (bilateral test).

## Results

Out of 178 children or adults with CHD undergoing CMR during the study period, 51 (29%) were included in the analysis. The main characteristics of the study population are shown in Table 3 .

**Table 3:** – Characteristics of the study population (n=51) and their diagnoses

Characteristics	
Male sex	30 (58%)
Weight (Kg) (SD)	33.0 ± 16.7
Body surface area (m2) (IQR)	1.0 (0.8-1.4)
Age at the time of surgical treatment (years) (IQR)	1.3 (0.8-2.4)
Age on the day of CMR (years) (IQR)	9.2 (6.9-13.3)
Age on the day of echocardiogram (years) (IQR)	9.0 (6.8-13.0)
Time between surgical treatment and CMR (years) (IQR)	7.2 (5.4-11.1)
Interval between CMR and echocardiogram (days) (IQR)	124 (70-188)
**Main diagnoses**	
Tetralogy of Fallot	35 (68%)
Pulmonary valve stenosis	6 (12%)
Common arterial trunk	3 (6%)
Pulmonary atresia with ventricular septal defect	2 (4%)
Tetralogy of Fallot with absent pulmonary valve	2 (4%)
Congenital pulmonary insufficiency	1 (2%)
Atrioventricular septal defect with pulmonary stenosis	1 (2%)
Pulmonary atresia with intact interventricular septum	1 (2%)

*IQR: interquartile range; CMR: cardiac magnetic resonance.*

### Echocardiographic findings

The dimensions of the RV were classified as abnormal in 96% of the cases, with mild dilation in 16%, moderate dilatation in 43% and severe dilatation in 37%. Had the echocardiographers considered only the right ventricular end-diastolic diameter RVEDD Z-score, there would be worsening in classification in 42 patients (82%), no changes in five patients (10%) and a better or more appropriate classification in four patients (8%) compared to CMR. Right ventricular dysfunction was reported in 20% of cases, being mild in 10%, moderate in 8% and severe in 2%. Out of the 51 patients, 29 (57%) had values of TAPSE, FAC or TDI in their reports, and among these 29 patients, 18 (62%) had measurements below the normal range. Despite this finding, nine of these 18 patients were classified as having normal systolic RV function. The agreement between these echocardiographic indices of RV systolic function and CMR right ventricular EF was 38%. Had the echocardiographer considered only these abnormal indices, disregarding the eyeball method, there would be misclassifications in nine more cases and a correct classification in five more cases. Echocardiographic measurements are presented in Table 4 . PI was mild in 2%, moderate in 2% and severe in 94%; in one case, PI was not classified. In none of the cases, LV systolic dilation or dysfunction was observed ( Table 5 ).

**Table 4:** – Echocardiographic measurements of the study patients

Parameters	Values
RV end-diastolic diameter (mm)	30.5 ± 7.48
RV end-diastolic diameter Z-score	2 (1.48-2.53)
TAPSE (mm) (n = 15)	17.9 ± 3.59
TAPSE Z-score	-2.3 (-3.28 a -0.94)
FAC (%) (n = 7)	41 ± 12.3
RV peak S´ cm/s (n = 23)	9.1 ± 1.89
RV peak S´ cm/s Z-score	-2.4 (-3.22 a -1.69)
RV diameter / LV diameter (mm)	0.8 (0.71-0.99)

*RV: right ventricular; TAPSE: tricuspid annular plane systolic excursion; fractional area change: FAC); RV peak S´: S’ wave peak systolic velocity of the right ventricle CMR findings.*

**Table 5:** – Comparison between two-dimensional echocardiogram (2D ECHO) and cardiac magnetic resonance (CMR) findings

			Normal	Mild dilation	Moderate dilation	Severe dilation
DIMENSION	RV	ECHO	2 (4%)	8 (16%)	22 (44%)	19 (38%)
		CMR	3 (6%)	3 (6%)	6 (12%)	39 (76%)
	LV	ECHO	51 (100%)	0	0	0
		CMR	38 (76%)	5 (10%)	3 (6%)	5 (10%)
			**Normal**	**Mild dysfunction**	**Moderate dysfunction**	**Severe dysfunction**
FUNCTION	RV	ECHO	41 (80%)	5 (10%)	4 (8%)	1 (2%)
		CMR	30 (59%)	15 (29%)	4 (8%)	2 (4%)
	LV	ECHO	51 (100%)	0	0	0
		CMR	35 (69%)	14 (27%)	1 (2%)	1 (2%)
			**None**	**Mild**	**Moderate**	**Severe**
PULMONARY INSUFFICIENCY		ECHO	0	1 (2%)	1 (2%)	48 (96%)
		CMR	1 (2%)	8 (16%)	32 (64%)	9 (18%)

*RV: right ventricle; LV: left ventricle.*

### CMR findings

The examination was performed under anesthesia in 16 patients (31%). The RVEDVi varied between 84.7 and 275.6 mL/m^2^ , which corresponded to an average Z-score of +5.6 ± 2. Three patients (6%) had a RVEDVi Z-score within normal limits (91.4 ± 7.9 mL/m^2^ ), another three (6%) presented mild dilation (110.6 ± 4.8 mL/m^2^ ), six (12%) moderate dilation (120 ± 10.9 mL/m^2^ ) and 39 (76%) severe dilation (151.8 ± 31.5 mL/m^2^ ). The LVEDVi varied between 66.4 and 155.2 ml/m^2^ ( Table 6 ). Dilation of the left ventricle was observed in 25% of patients and LV systolic dysfunction was found in 31% ( Table 5 ). Of the 16 patients with LV dysfunction, in 12 (75%) the exam was performed under anesthesia.

**Table 6:** – Main cardiac magnetic resonance findings in the study patients (n=51)

Parameters analyzed	Values
RV end-diastolic volume indexed (ml/m^2^)	142.1 ± 33.4
RV end-systolic volume indexed (ml/m^2^)	76.1 ± 27.4
RV ejection fraction (%)	46.9 ± 10.2
Pulmonary insufficiency (L/min/m^2^)	1.5 (1.01-1.99)
Pulmonary insufficiency (%)	31.1 ± 10.7
LV end diastolic volume indexed (mL/m^2^)	87.4 ± 18.5
LV end systolic volume indexed (mL/m^2^)	42.4 ± 12.8
LV ejection fraction (%)	53.6 ± 8.4%
RV volume / LV volume ratio	1.6 (1.43-1.86)

*RV: right ventricle; LV: left ventricle.*

## Agreement analysis between 2D ECHO and CMR

### Dimension of the right ventricle

A significant agreement was observed between the methods for the classification of right ventricular dimensions (Kappa 0.19; 95% CI 0.05 to 0.33, p 0.004), although the degree of this agreement was low. The difference in classification was of one degree in 74% of the discordant cases, with an underestimation of the dimensions of the RV by ECHO in 22 cases (43%) and overestimation in five cases (10%) ( Figure 1 ). Of the 39 cases of severe dilation, ECHO made the correct diagnosis in 17 (44%). The sensitivity of the ECHO to detect moderate or severe dilations of the RV was 87% and the specificity was 67%.

**Figure 1:** – *Graph comparing the classification of RV dimension by CMR and ECHO for each patient.*

### Right ventricular systolic function

There was a weak agreement between the methods for classification of the RV function (Kappa 0.16; 95% CI -0.01 to +0.34; p 0.034). The difference in classification was of one degree in 86% of the discordant cases, with the RV function overestimated by ECHO in 15 cases (29%) and underestimated in six cases (12%) ( Figure 2 ). The two cases with severe RV dysfunction were not detected by ECHO: one was classified as normal and the other as moderate dysfunction.

**Figure 2:** – *Graph comparing the classification of RV systolic function by CMR and ECHO for each patient.*

### Pulmonary insufficiency

The degree of agreement between the methods for classification of PI was not statistically significant (Kappa 0.014; 95% CI -0.03 to +0.06, p 0.27). There was disagreement in 80% of the cases, with ECHO tending to overestimate the degree of PI. There was disagreement above one degree in 20% of the cases ( Figure 3 ).

**Figure 3:** – *Graph comparing the classification of pulmonary insufficiency by CMR and ECHO for each patient.*

### Left ventricular systolic function and dimension

ECHO did not identify any of the 16 cases (31%) of LV dysfunction and none of the 13 cases (25%) of LV dilation, preventing the assessment of agreement between the methods. Of the 16 cases of LV dysfunction, 10 (62%) also had RV dysfunction and only one had normal-sized RV.

## Discussion

Residual PI and remodeling of the RV over time are important elements in the long-term course of patients who have undergone RV outflow tract repair in childhood. Decision making regarding the time to perform a new surgical intervention in these cases is still challenging. Clinical and electrocardiographic criteria, in addition to findings of image examinations, such as ECHO and CMR, are used to assist in this decision, seeking to determine the optimal time to perform surgical treatment.

The 2D ECHO is the most used imaging method to monitor the course of these patients, guiding, in most cases, the decision-making. Given the difficulties in performing a 2D echocardiographic assessment of the RV with objective parameters, qualitative assessment (eyeball) of the dimensions and function of the RV is still widely used. However, the accuracy of this assessment is questionable and still motivates further studies. Our study sought to assess the accuracy of 2D ECHO, performed during the outpatient routine, in quantifying the dimensions and function of the RV and the degree of PI. We selected patients with CHD at risk to develop RV dilation and dysfunction and PI.

We observed that there was a certain, albeit weak, agreement between ECHO and CMR for the classification of right ventricular dimensions. This finding is similar to that found by Lai et al.,^4^ when they applied the guidelines for the assessment of the RV using 2D ECHO in normal patients, in patients with atrial septal defects and in patients who had underwent tetralogy of Fallot repair. In their article, the authors postulate that 2D ECHO inaccuracies are inherent to the application of linear or area measurements in a complex geometry chamber. Puchalski et al.,^18^ in a study with a design similar to ours, obtained similar results and emphasized the great interobserver variability as a potential source of disagreement between ECHO and CMR. It is suggested that the adding of objective to subjective measures would increase the agreement between the methods.^7 , 19^ In this sense, Greutmann et al.^20^ observed, in an adult population, that the linear measurement with the best correlation with the RV diastolic volume was the RV area in the four-chamber apical view. By measuring this area, Alghandi et al.^21^ were able to discriminate patients with RVEDVi > 170 ml/m^2^ . More recent studies have investigated the use of 3D ECHO to assess the RV and, even so, it is generally observed underdimensioning of the RV^7 , 22 - 24^ is explained by its incomplete visualization, especially in cases of severe RV dilation, when the cardiac apex is dislocated and difficult to access by ECHO inspection. In our study, although we analyzed a young population, 86% of the patients had moderate or severe dilation of the RV. This could explain the lack of accuracy, if we consider that the bigger the RV, the greater the difficulties in evaluating it. However, in our study, 2D ECHO was able to detect 96% of cases of moderate and / or severe dilation, which may serve the purpose of screening patients who would need to undergo CMR.

Regarding RV systolic function, our results are in line with those of the study by Puchalski,^18^ who reported a poor agreement between 2D ECHO and CMR. However, the degree of disagreement between the stratification classes was only of one grade in 86% of the cases, and this difference occurred mainly in the zone between mild dysfunction and normal function. Several echocardiographic methods have been used to assess right ventricular function, including TAPSE, FAC, TDI, Tei index, among others. Although some of them are reproducible, none of them has shown a strong correlation with the right ventricular EF in the pediatric population, especially in patients with CHD.^3 , 7 - 10 , 25 - 27^ Methods with unidimensional measurements, such as TAPSE, or bidimensional measurements such as FAC, do not take into account the complex geometry of the RV. These two methods do not consider the RV outflow tract, for example, commonly compromised in patients undergoing tetralogy of Fallot correction.^21^ In addition, there are other factors influencing these measurements, such as previous pericardiectomy, preload, ultrasound beam angulation, echocardiographic window quality, LV function, etc. In the study by Mercer-Rosa et al.^16^ TAPSE was not correlated to right ventricular EF by CMR, nor to the peak VO_2_. The same author, in 2012, observed a weak correlation between the tissue Doppler of the right ventricular free wall and the right ventricular EF by CMR, and the lack of correlation between the Tei index and the right ventricular EF by CMR in patients post-tetralogy of Fallot repair.^5^ In the study by Leong et al.^28^ in an adult population with heart failure, right ventricular speckle tracking strain showed the strongest correlation (r = 0.77) with CMR right ventricular EF, compared with other techniques including TAPSE, FAC and TDI. However, one has to consider that this population usually does not present with severe RV dilatation. 3D ECHO is perhaps the method that best correlates with CMR,^29^ but it is still far from becoming a method as available as the 2D ECHO, especially in the young population.^30^ In practice, many services end up using qualitative evaluation (eyeball) of the right ventricular function, combined with some of the aforementioned indices to corroborate the final impression. The assessment of the function of the RV by 2D ECHO, therefore, becomes an examiner-dependent method and subject to different interpretations.

With regard to the degree of PI, we did not find any agreement between ECHO and CMR in our study. In 38 patients (75% of the sample), PI was classified as severe by ECHO, but as moderate or mild by CMR. In other words, ECHO overestimated the degree of PI in most cases, a data similar to that observed by Mercer-Rosa et al.^5^ In that study,^5^ many patients with mild PI by CMR were classified as having moderate or severe PI by ECHO, when using the diastolic VTI / systolic VTI ratio obtained in the pulmonary trunk. In turn, Renella et al.^31^ observed that ECHO had great sensitivity in identifying cases of severe PI in different methods used and recommend the evaluation of Doppler curves in the pulmonary branches, instead of in the pulmonary trunk, to increase the specificity of the method. In practice, the literature has shown that ECHO is able to discriminate cases of mild PI from cases of moderate or severe PI, which is corroborated by our findings.

LV dysfunction has been described in patients with RV disease repair and linked to major adverse events.^32 - 37^ Therefore, LV assessment cannot be neglected. In our sample, although the 2D ECHO showed normal LV function in all patients, CMR identified LV dysfunction in 31% of cases, which indicates the limitations of 2D ECHO in assessing LV function in this situation. Of the patients with LV dysfunction, 62% also had some degree of RV dysfunction, which may be related to ventricular interdependence.^38 , 39^ In our view, the evaluation of LV function in cases of RV dilation or dysfunction is more difficult to the ECHO due to the paradoxical movement of the interventricular septum and the LV dislodgement by a dilated or dysfunctional RV, making its visibility more difficult.

### Study limitations

This was a retrospective, single-center study with a limited number of patients. The possibility of inclusion bias should be considered, since the decision to submit the patient to CMR is often made after analysis of ECHO findings. Analysis of ECHO results was based on the reports only, without image review. Considering that five echocardiographers performed the exams, there is the possibility of intraobserver and interobserver disagreements. Also, time interval between ECHO and CMR was relatively large. Although there was no surgical or percutaneous interventional procedure between ECHO and CMR, ideally the exams should have been performed within 24 hours of each other. The use of Z-score of ventricular measurements obtained by CMR is not a common approach in the literature, and the classification range, as well as the clinical impact of these measurements, need to be better evaluated in future studies.

## Conclusions

In our practice, and considering the study limitations, 2D ECHO showed a low agreement with CMR regarding right ventricular dimensions and systolic function, and degree of PI. In general, ECHO underestimated the dimensions of the RV and overestimated the function of the RV and the degree of PI. We understand that the incorporation of more objective echocardiographic parameters is necessary for a better agreement with the CMR. However, the real impact of this analysis in relation to clinical decision-making is still uncertain.
